# Gut Microbiota Dysbiosis Mediates ^125^I‐Induced Radiation Colitis via Host Transcriptional Regulation

**DOI:** 10.1002/iid3.70523

**Published:** 2026-09-15

**Authors:** Pingping Liu, Xiong Zeng, Wenjing Liu, Huichao Xie, Yongkun Fang, Erwan Yang, Xiaoqi Tang, Chenglin Fan, Yihui Chen

**Affiliations:** ^1^ Department of Medical Imaging Sichuan Corps Hospital of PAP Leshan China; ^2^ Department of General Surgery, Xinqiao Hospital Army Medical University Chongqing China; ^3^ Department of General Surgery The First Mobile Corps Hospital of PAP Dingzhou China; ^4^ Department of Laboratory Medicine The First Medical Center of Chinese PLA General Hospital Beijing China

**Keywords:** iodine‐125 (^125^I) seed therapy, microbial dysbiosis, radiation colitis

## Abstract

**Background:**

Iodine‐125 (^125^I) seed therapy is recognized for treating abdominal solid tumors with minimal adjacent tissue damage but may induce radiation colitis when its implantation near colonic tissues, leading to a poor prognosis. Nevertheless, the underlying pathogenesis is not well understood.

**Objective:**

This research aims to investigate the role of gut microbiota and host immune‐transcriptional responses in the pathogenesis of ^125^I‐induced radiation colitis.

**Methods:**

We implanted ^125^I seeds intraperitoneally in mice and assessed colonic damage via histology, TUNEL staining, cytokine assays, and tight‑junction protein measurements. Fecal microbiota were profiled by 16S rRNA sequencing, and colonic transcriptomes by RNA‑seq. Multi‑omics integration (Spearman correlation, KEGG, RDA, Procrustes) was used to link microbial taxa, differentially expressed genes, and pathological features.

**Results:**

^125^I exposure caused mucosal necrosis, lowered ZO‐1 and occludin levels, and raised IL‐1α, IL‐1β, IL‐6, and TNF‐α. 16S rRNA sequencing revealed altered microbial β‐diversity and dysbiosis, characterized by reduced *Lachnospiraceae*, *Mucispirillum*, and *Desulfovibrio*, and increased *Clostridium methylpentosum* and *Bifidobacterium*. RNA‐seq analysis revealed differentially expressed genes such as Angptl7, Apoh, C7, and Ccl7. Integrated analyses, including Spearman correlation, KEGG enrichment, redundancy analysis, and Procrustes analysis, confirmed strong associations between certain microbes, host gene expression, and pathological phenotypes.

**Conclusion:**

Our findings indicate that ^125^I irradiation is associated with gut dysbiosis and concurrent host immune‑transcriptional reprogramming, which may jointly contribute to radiation colitis. Although these results lay a foundation for microbiota‑targeted interventions, the causative role of microbial changes remains to be confirmed by functional experiments.

## 1 Introduction

Brachytherapy utilizing ^125^I seed has become an important therapeutic option for abdominal solid tumors, including those of the prostate, pancreas, liver, colorectum, and gynecologic organs [1, 2]. This approach is advantageous due to its capacity to deliver continuous, low‐dose‐rate gamma radiation that precisely targets tumors while minimizing damage to surrounding healthy tissues. Nevertheless, given the proximity of abdominal malignancies to intestinal structures, radiation exposure often leads to unintended damage, which radiation colitis is one of the most common and serious complications [3, 4, 5]. This condition is characterized by persistent inflammation, compromised mucosal barrier integrity, and impaired bowel function, all of which significantly diminish patients’ quality of life and adversely affect treatment outcomes [6, 7]. However, the mechanisms underlying ^125^I‐induced radiation colitis remain incompletely elucidated.

Traditional perspectives primarily attribute the pathology to direct radiation‐induced damage to intestinal epithelial cells and the consequent aberrant DNA damage responses [8]. Recently, there has been a shift in focus towards the gut microbiota, now recognized as a central mediator of intestinal homeostasis and a critical regulator of mucosal immunity [9]. Dysbiosis has been strongly linked to the pathogenesis of inflammatory colonic diseases, such as ulcerative colitis and Crohn's disease, where the interplay between microbial imbalance and immune dysregulation is well‐established [10]. Importantly, the gut microbiota is highly sensitive to ionizing radiation, and recent studies have demonstrated that radiation can significantly alter microbial composition, with such disruptions exacerbating radiation‐induced intestinal injury [6, 11]. This suggests that the microbiota may serve as a key regulator between radiation exposure and the host inflammatory response. Nonetheless, the majority of current research has concentrated on high‐dose‐rate external irradiation models, leaving the dynamic changes in the gut microbiota induced by continuous low‐dose‐rate ^125^I irradiation and its specific role in the pathogenesis of colitis insufficiently explored. Our study provides critical insights into the dynamics of the gut microbiota after ^125^I irradiation and underscores its pivotal function in regulating colonic inflammation and immunity.

Accordingly, this study was designed to investigate whether ^125^I seed implantation leads to radiation colitis through the disruption of gut microbial ecology. By elucidating the role of the gut microbiota in this process, our research not only provides new insights into the microbiological mechanisms underlying ^125^I‐induced colonic injury but also underscores the immunological dimensions of this complication. We believe these findings will contribute to the development of innovative therapeutic strategies that target the modulation of the gut microbiome to prevent or mitigate radiation colitis.

## 2 Results

### 2.1 Colonic Tissue Damage and Release of Pro‐Inflammatory Cytokines in Mice Following ^125^I Exposure

**Figure 1:**
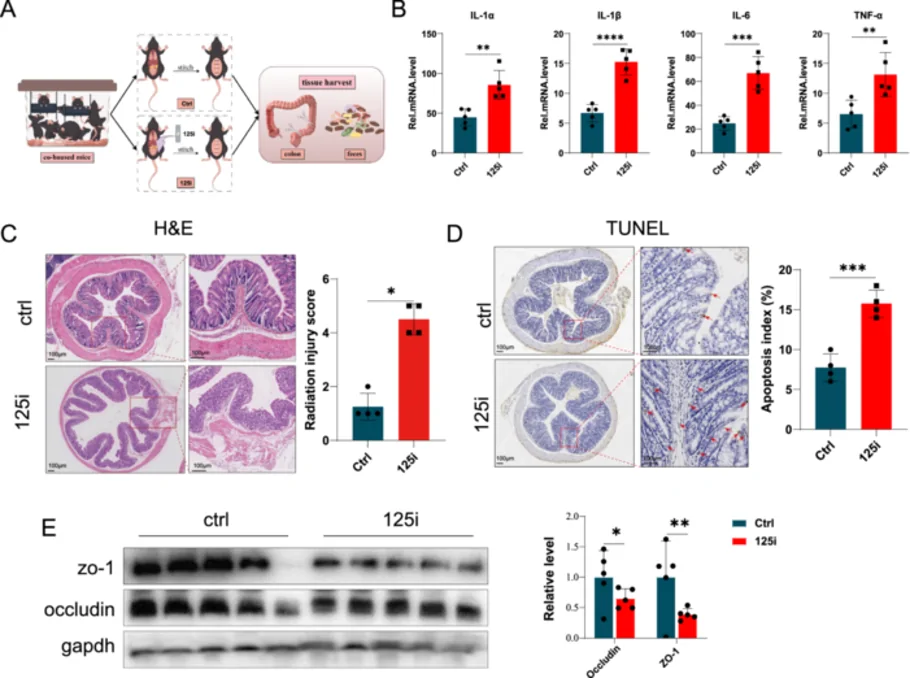
Implantation of ^125^I seed in the abdominal cavity of mice induces colonic tissue damage. (A) C57BL/6 mice were co‐housed and randomly assigned to two groups: a control group, which underwent sham surgery, and an experimental group, which received intraperitoneal implantation of ^125^I seed. Two weeks post‐procedure, colon tissue and fecal samples were collected from all mice. (B) Quantitative polymerase chain reaction (qPCR) analysis revealed significantly elevated mRNA expression levels of IL‐1α, IL‐1β, IL‐6, and TNF‐α in the colonic tissue of the ^125^I group compared to the control group. (C) Hematoxylin and eosin (H&E) staining was performed on the colon tissue, and damage was assessed using radiation injury score. Scale bar = 100 μM. (D) Terminal deoxynucleotidyl transferase dUTP nick end labeling (TUNEL) staining was conducted to analyze the colon tissue, with apoptosis quantified by calculating the apoptotic index. Scale bar = 100 μM. Red arrows indicate apoptotic cells. (E) Western blot analysis was employed to assess the expression levels of the tight junction proteins ZO‐1 and occludin in the colon tissue, with quantitative analysis performed using ImageJ software. The values were presented as the mean ± SD in Figure 1 (B, E) (*n* = 5) and Figure 1 (C, D) (*n* = 4); *p < 0.05; **p < 0.01; ***p < 0.001 compared with the control group.

To assess the potential induction of intestinal injury by ^125^I seed implantation, we surgically introduced ^125^I sources into the abdominal cavities of mice and subsequently collected colon tissue and fecal samples for comprehensive analysis (Figure 1A). Throughout the two‑week experimental period, mice in the ^125^I‑treated group exhibited soft stools or mild diarrhea compared to controls, but no signs of bloody stool, significant weight loss, or altered behavior were observed. Our results demonstrated a significant upregulation in the expression of pro‐inflammatory cytokines, including IL‐1α, IL‐1β, IL‐6, and TNF‐α, in colon tissue following ^125^I irradiation, with all observed changes reaching statistical significance (Figure 1B). Histological examination using H&E staining revealed structural impairments in the ^125^I‐treated group, characterized by widening of the submucosal space, detachment of the mucosal layer from the submucosa, extension of these pathological alterations along both sides of the colonic mucosa, and pronounced infiltration of inflammatory cells (Figure 1C). Concurrently, TUNEL assays detected apoptotic cells within the colonic mucosa, corroborating the occurrence of epithelial cell necrosis post‐^125^I exposure (Figure 1D). Further investigations indicated a marked reduction in the expression of tight junction proteins ZO‐1 and occludin within colonic epithelial cells, suggesting a compromised barrier function (Figure 1E). Collectively, these findings indicate that ^125^I irradiation causes significant disruption to colonic architecture, facilitates the extensive release of pro‐inflammatory cytokines, and ultimately plays a role in the pathogenesis of radiation‐induced colitis.

### 2.2 Exposure to ^125^I Radiotherapy Resulted in a Significant Restructuring of the Microbial Community Diversity in Mice

**Figure 2:**
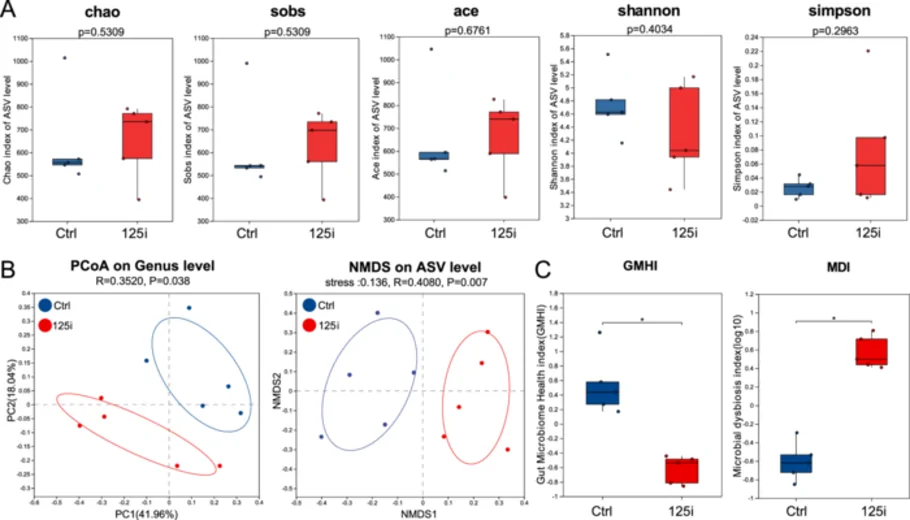
Exposure to ^125^I radiotherapy significantly altered the diversity of the microbial community in mice. (A) Alpha diversity indices at the genus level assess species richness and diversity within a sample. The chao1 and ace indices measure species richness, with higher values indicating more species. The sobs index shows the actual number of species observed. The Shannon index considers both richness and evenness, reaching its peak when both are high, while the Simpson index reflects the likelihood of two random individuals being the same species, with higher values indicating less richness. Alpha diversity indices were compared between the control and ^125^I‐treated groups using the Wilcoxon rank‐sum test. (B) Beta diversity indices at the genus level, based on PCoA and NMDS, use Bray–Curtis distance algorithms. PCoA: *R* = 0.3520, *p* = 0.038. NMDS: Stress = 0.136, *R* = 0.4080, *p* = 0.007. Significant differences in microbial community structure between groups were evaluated using analysis of similarities (ANOSIM) with 999 permutations. (C) The intestinal microbiota health index (GMHI) suggests a healthier microbial community in the control group, whereas the Microbial dysbiosis index (MDI) indicates significant dysbiosis in the ^125^I group. The values were presented as the mean ± SD in Figure 2 (A–C) (*n* = 5); *p < 0.05; **p < 0.01; ***p < 0.001 compared with the control group.

To assess whether significant changes occur in the intestinal microbiota following ^125^I seed implantation, we conducted 16S rRNA sequencing on fecal samples. In the context of alpha diversity analysis, the chao1 and ace indices evaluate species richness within the sample, with higher values indicating a greater variety of species present. The sobs index represents the actual number of species observed in the sample. Both the Shannon and Simpson indices measure species diversity; the Shannon index accounts for both species richness and the evenness of species distribution. When both richness and evenness are high, the Shannon index reaches its maximum value. Conversely, the Simpson index indicates the probability that two individuals randomly selected from the sample belong to the same species, with a higher value suggesting lower species richness. The results indicate that while there is a variation in alpha diversity levels between the ^125^I group and the control group, this difference is not statistically significant (Figure 2A). However, the two groups exhibited significant differences in beta diversity. Principal co‐ordinates analysis (PCoA) showed distinct separation between the groups, indicating a significant difference in species composition (*R* = 0.352, *p* = 0.038). Non‐metric Multidimensional Scaling (NMDS) confirmed the reliability of these results with a low stress value (Stress < 0.2, Figure 2B). Additionally, The GMHI and MDI values, analyzed as exploratory metrics, suggested a relatively healthier microbial community in the control group and a trend toward dysbiosis in the ^125^I‑treated group (Figure 2C).

### 2.3 Analysis of Intestinal Microbiota Composition and Biomarker Genus Following ^125^I Radiotherapy

**Figure 3:**
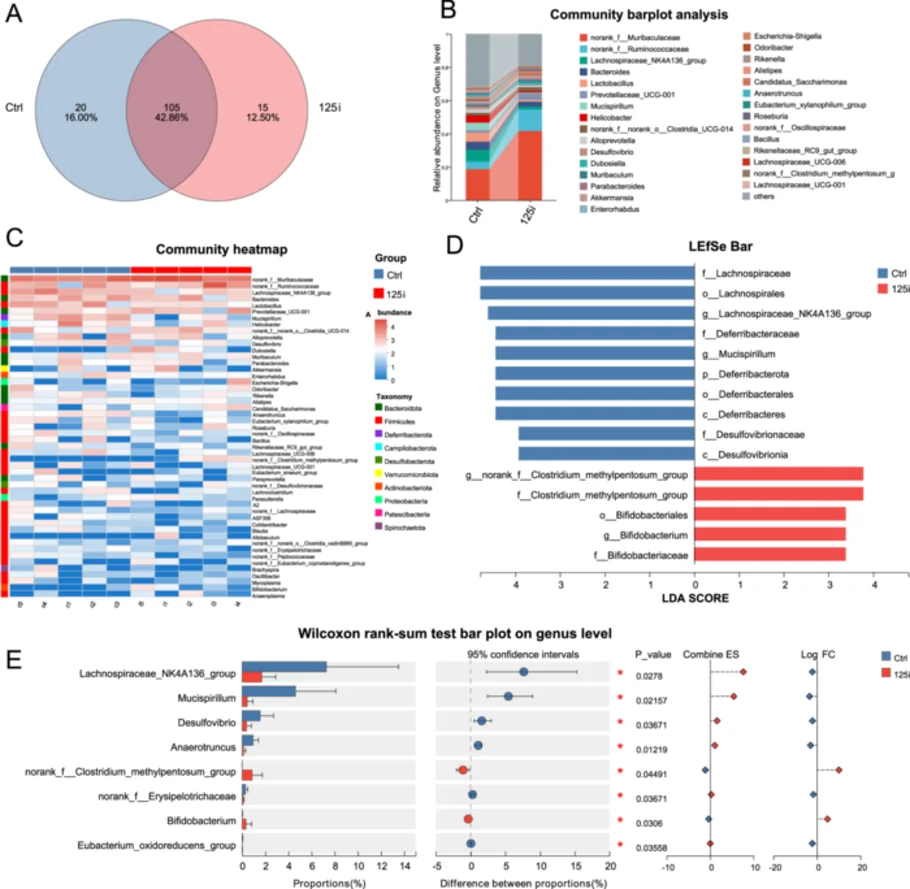
Analysis of intestinal microbiota composition and biomarker genus following ^125^I radiotherapy. (A) A Venn diagram analysis showed that the control group had 125 genera, including 20 unique ones, while the ^125^I‐irradiated group had 120 genera, with 15 unique. (B) A community bar plot indicated significant taxonomic changes after irradiation, with increased *norank_f_Muribaculaceae* and *norank_f_Ruminococcaceae*, and decreased *Lachnospiraceae_NK4A136_group* in the ^125^I‐treated group. (C) A heatmap illustrated species abundance patterns for the top 50 most abundant genera, highlighting distinct microbiota profiles for each sample. (D) LEfSe analysis identified key features from phylum to genus, using LDA scores to measure the impact of each taxon. The family *Lachnospiraceae* (along with its corresponding order and genus‑level representatives) was enriched in the control group, whereas the C. methylpentosum group (at the family level) and the family Bifidobacteriaceae were increased in the ^125^I group. (E) The Wilcoxon rank‐sum test was used at the genus level to determine *p* values, revealing that *Lachnospiraceae_NK4A136_group*, *Mucispirillum*, and *Desulfovibrio* were the most significantly altered after irradiation. The values were presented as the mean ± SD in Figure 3 (A–E) (*n* = 5); *p < 0.05 compared with the control group.

To further elucidate the structural alterations in intestinal microbiota induced by ^125^I irradiation, we performed a Venn diagram analysis to discern shared and unique microbial taxa between the experimental groups. The analysis revealed that, at the genus level, the control group harbored ^125^ genera, including 20 unique taxa, whereas the ^125^I‐irradiated group comprised 120 genera, with 15 being unique to this group (Figure 3A). A bar plot depicting community composition demonstrated significant changes in taxonomic abundance post‐irradiation. Notably, the relative abundances of *norank_f_Muribaculaceae* and *norank_f__Ruminococcaceae* showed an increasing trend in the ^125^I‐treated group, while the abundance of *lachnospiraceae_NK4A136_group* was markedly decreased (Figure 3B). A heatmap was constructed to visualize species abundance patterns across samples, highlighting distinct microbiota profiles specific to each sample (Figure 3C). LEfSe analysis was employed to identify discriminative features from the phylum to genus level, with LDA scores quantifying the effect size of each differential taxon. Notably, the family *Lachnospiraceae* were enriched in the control group, whereas the families *Christensenellaceae* and *Bifidobacteriaceae* exhibited significant increases in the ^125^I group (Figure 3D). This observation suggests a potential link to radiation‐induced dysbiosis and associated pathological processes. To validate these differentially abundant taxa, Wilcoxon rank‐sum tests were employed. Following abundance‐based filtering, the genera *Lachnospiraceae_NK4A136_group*, *Mucispirillum*, and *Desulfovibrio* emerged as the most significantly altered post‐irradiation (Figure 3E). These findings highlight their potential roles as biomarkers or functional contributors in studies of radiation‐related microbiome alterations.

### 2.4 Analysis of Links Between Colonic Damage, Inflammatory Cytokines, and Microbiota Changes in Mice

**Figure 4:**
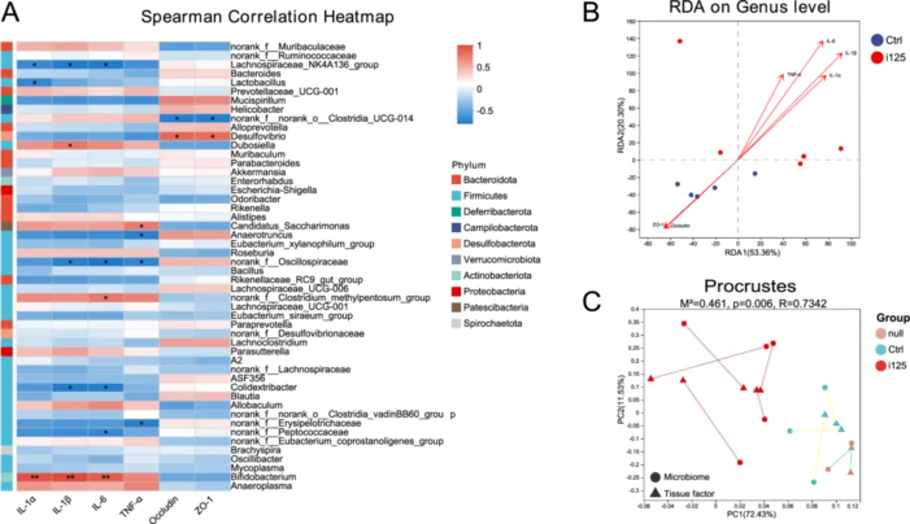
Analysis of colonic damage, inflammatory cytokines, and microbiota changes in mice revealed significant correlations. (A) A Spearman correlation heatmap indicated negative correlations between pro‐inflammatory cytokines (IL‐1α, IL‐1β, IL‐6, TNF‐α) and microbial taxa such as *Lachnospiraceae_NK4A136_group*, *Lactobacillus*, and *norank_f_Oscillospiraceae*. In contrast, *Bifidobacterium* positively correlated with these cytokines. Additionally, *norank_f_norank_o_Clostridia_UCG‐014* negatively correlated with intestinal barrier proteins, while *Desulfovibrio* showed a positive correlation. (B) Redundancy analysis (RDA) identified IL‐1β and IL‐6 as key cytokines linked to microbial community variations, suggesting their biomarker potential. Blue points represent the control group, red points the ^125^I‐treated group. Red arrows show clinical factors, with arrow length indicating their influence on species distribution. Acute, obtuse, and right angles between arrows indicate positive, negative, and no correlations, respectively. (C) Procrustes analysis revealed a significant correlation between microbiota composition and histopathological factors. In the figure, colors denote experimental groups. Each line connects a sample's microbiome ordination (solid square) to its histopathological factor ordination (solid circle). The line's length represents Procrustes residuals, indicating dissimilarity between the datasets; shorter lines mean greater consistency. The values were presented as the mean ± SD in Figure 4 (A–C) (*n* = 5); *p < 0.05 compared with the control group.

In this study, we observed both colonic injury and intestinal microbiota dysbiosis following the implantation of ^125^I seed. To determine whether the microbial alterations were associated with colonic damage, we conducted a spearman correlation analysis between inflammatory cytokines and tight junction protein levels and the top 50 altered microbial taxa. The analysis revealed potential associations: several microbial taxa, including *Lachnospiraceae_NK4A136_group*, *Lactobacillus*, and *norank_f_Oscillospiraceae*, showed negative correlation trends with pro‐inflammatory cytokines, whereas *Bifidobacterium* exhibited positive correlation trends with these inflammatory factors. Additionally, *norank_f_norank_o_Clostridia_UCG‐014* showed negative correlation trends with intestinal barrier proteins, while *Desulfovibrio* showed positive correlation trends (Figure 4A). Redundancy analysis (RDA) further identified IL‐1β and IL‐6 as the cytokines most strongly associated with variations in the microbial community, as evidenced by their longest vector lengths in Figure 4B, suggesting their potential as key biomarkers in this context (Figure 4B). To assess the overall concordance between microbiota composition and the measured host parameters (four cytokines and two tight junction proteins), we performed a Procrustes analysis. And the result revealed a significant correlation between the two datasets, wherein shorter connecting lines indicated greater consistency in their multivariate configuration (Figure 4C).

### 2.5 RNA‐Seq Transcriptomics Analysis of Mouse Colonic Tissue Following ^125^I Exposure

**Figure 5:**
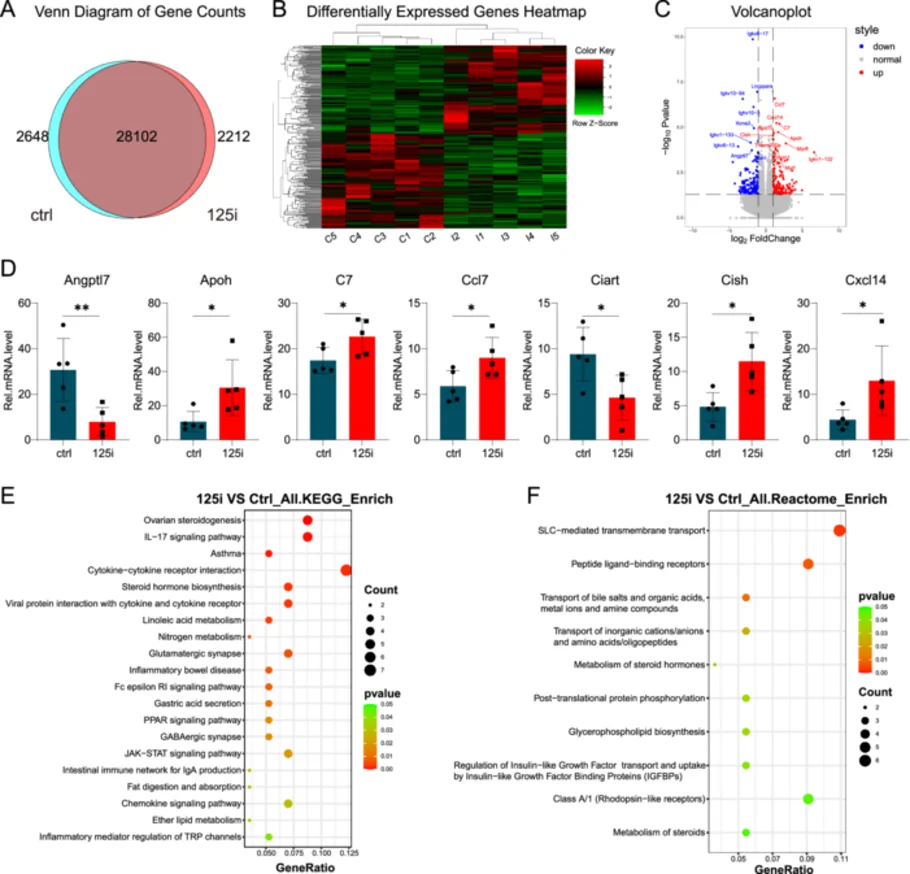
RNA‐Seq analysis of mouse colonic tissue following ^125^I exposure. (A) A Venn diagram showed 28,102 shared genes between the two experimental groups, with 2,648 unique to the control and 2,212 unique to the ^125^I‐treated group. (B) Cluster analysis of differentially expressed genes (DEGs) revealed distinct expression patterns, with upregulated genes in red and downregulated genes in green. (C) A volcano plot identified the top 20 DEGs, including angptl7, apoh, C7, and so forth, with upregulated genes in red and downregulated ones in blue. (D) qPCR method was used to validate the differential expression of selected genes, including angptl7, apoh, c7, ccl7, ciart, cish, and cxcl14 in colon tissue. (E) KEGG pathway analysis identified the cytokine–cytokine receptor interaction and IL–17 signaling pathways as the most enriched, showing the highest gene ratios and significant statistical differences. (F) Reactome functional enrichment analysis revealed that these DEGs were predominantly associated with SLC‐mediated transmembrane transport and peptide ligand‐binding receptors. The values were presented as the mean ± SD in Figure 5 (A–F) (*n* = 5); *p < 0.05 compared with the control group.

To elucidate the principal molecular mechanisms underlying ^125^I radiation‐induced colitis and its correlation with alterations in intestinal microbiota, we conducted RNA‐Seq analysis on colonic tissue samples. A Venn diagram of gene counts identified 28,102 genes common to both experimental groups, with 2648 genes unique to the control group and 2212 genes unique to the ^125^I‐treated group (Figure 5A). Cluster analysis of differentially expressed genes (DEGs) revealed distinct gene expression patterns between the two groups, with upregulated and downregulated genes clearly separated (red and green, respectively)(Figure 5B). A volcano plot highlighted the top 20 most significantly DEGs such as angptl7, apoh, C7, and so forth. (Figure 5C), among which several were identified as belonging to the immunoglobulin variable region genes (Igkv/Ighv families), integral components of B cell receptors. Although the individual roles of these genes may be limited, their collective differential expression suggests a potentially significant involvement of B cells in the pathogenesis of ^125^I‐induced colitis. Subsequently, we validated the differential expression of selected genes, including angptl7, apoh, c7, ccl7, ciart, cish, and cxcl14 in colon tissue through qPCR method (Figure 5D), confirming their significant alteration following ^125^I treatment. Analysis using the kyoto encyclopedia of genes and genomes (KEGG) pathway identified the cytokine–cytokine receptor interaction and IL–17 signaling pathways as the most enriched, demonstrating the highest gene ratios and most significant statistical differences (Figure 5E). This indicates a substantial concentration of DEGs within these pathways. Furthermore, reactome functional enrichment analysis revealed that these DEGs were predominantly associated with SLC‐mediated transmembrane transport and peptide ligand‐binding receptors (Figure 5F), with both categories exhibiting significant enrichment.

### 2.6 Correlation Between DEGs and Altered Microbial Composition

**Figure 6:**
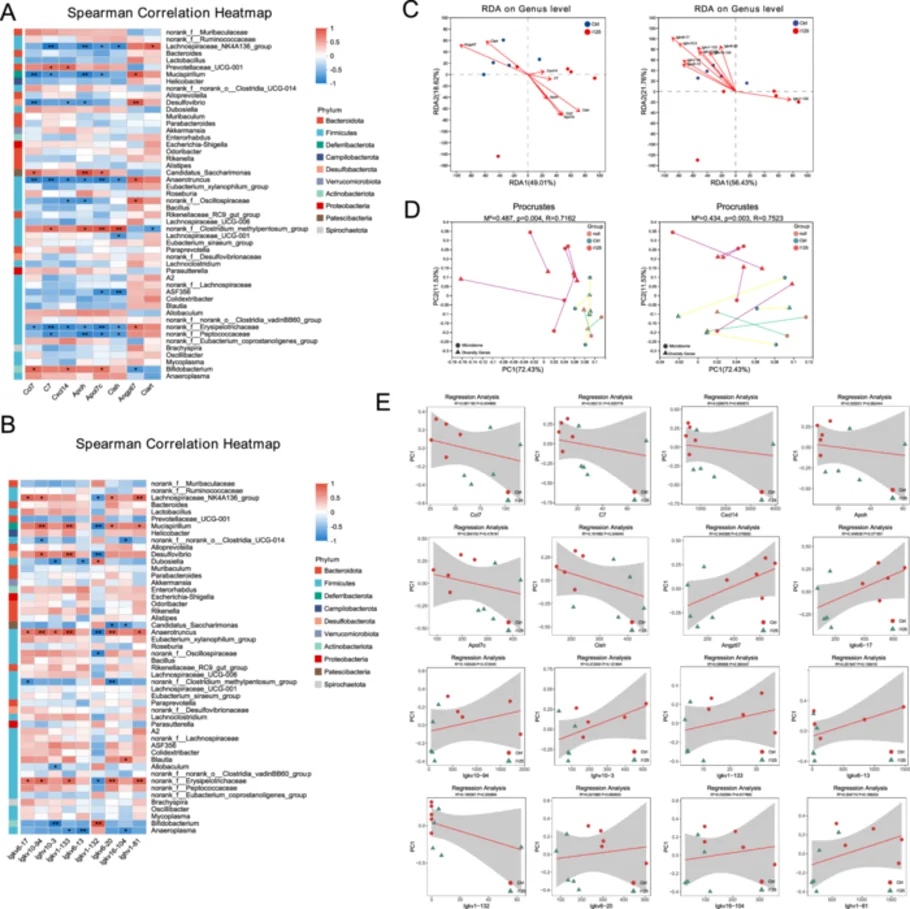
Correlation between DEGs and altered microbial composition. (A) A Spearman correlation heatmap revealed associations among ccl7, c7, cxcl14, apoh, apol7c, cish, angptl7, ciart and microbial alterations. (B) A Spearman correlation heatmap demonstrated associations among Igkv6‐17, Igkv10‐94, Ighv10‐3, Igkv1‐133, Igkv6‐13, Igkv1‐132, Igkv6‐20, Igkv16‐104, Ighv1‐81 and microbial alterations. (C) RDA identified angptl7, ciart, Igkv6‐17, and Igkv1‐132 as the genes most strongly correlated with variations in the microbial community, suggesting their potential as key biomarkers within this regulatory network. (D) Procrustes analysis was employed to assess the overall concordance between gut microbiota composition and the transcriptomic profile of colonic injury. The presence of shorter connecting lines in the Procrustes plot indicated a higher degree of similarity between the two datasets, reflecting coordinated variation. (E) Linear regression analysis between individual DEGs and beta diversity indices, evaluating the impact of specific genes on microbial community structure. A higher *R* ^2^ value indicated a more substantial contribution of the gene to the variance observed in species composition. The values were presented as the mean ± SD in Figure 6 (A–E) (*n* = 5); *p < 0.05 compared with the control group.

To further explore the association between DEGs and microbial alterations, we categorized the DEGs into two clusters and conducted a Spearman correlation analysis with the top 50 altered microbial taxa. The analysis suggested potential correlations between specific microbes, such as *Lachnospiraceae_NK4A136_group*, *Mucispirillum*, and *Desulfovibrio*, and the expression of these DEGs (Figure 6A,B). However, after FDR correction (q < 0.05), the strength of these associations was reduced; therefore, these findings should be interpreted as exploratory and hypothesis‐generating. The complete correlation data, including all *p* values and *q*‐values, are provided in Supplementary Tables. Subsequent RDA analysis identified angptl7, ciart, Igkv6‐17, and Igkv1‐132 as the genes most strongly correlated with variations in the microbial community, indicating their potential as key biomarkers within this regulatory network (Figure 6C). Additionally, procrustes analysis was utilized to evaluate the overall concordance between gut microbiota composition and the transcriptomic profile of colonic injury. The presence of shorter connecting lines in the procrustes plot suggested a higher degree of similarity between the two datasets, reflecting coordinated variation (Figure 6D). Finally, we performed a linear regression analysis between individual DEGs and beta diversity indices using the R lm() function to assess the impact of specific genes on microbial community structure. A higher *R* ^2^ value indicated a greater contribution of the gene to the variance observed in species composition, with 95% confidence intervals calculated for the fitted lines (Figure 6E).

## 3 Discussion

In this study, we developed a murine model for intraperitoneal ^125^I seed implantation and revealed that ^125^I irradiation exposure significantly disrupted the structural composition of the gut microbiota, evidenced by a notable decline in beneficial microbiota such as *Lachnospiraceae_NK4A136_group* and *Lactobacillus*. These microbial changes were associated with elevated levels of colonic inflammatory cytokines and compromised barrier function. Subsequent RNA‐Seq analysis identified several DEGs in the host, including Angptl7, Apoh, C7, Ccl7, Ciart, Cish, and Cxcl14, and highlighted key mechanistic pathways, particularly the cytokine–cytokine receptor interaction and IL–17 signaling pathways. Crucially, through correlation analysis, we elucidated significant interactions between the intestinal microbiota and these DEGs, constructing an integrated network that connects microbial alterations with host transcriptional responses. Collectively, these findings unveil a novel mechanism by which continuous low‐dose‐rate ^125^I irradiation facilitates the onset of radiation colitis.

Brachytherapy utilizing ^125^I seed has emerged as a minimally invasive and significant modality for the treatment of intra‐abdominal solid tumors, including prostate, pancreatic, and liver cancers [12, 13]. This technique is favored for its capacity to deliver continuous low‐energy gamma radiation, thereby establishing a high‐dose zone within the tumor while preserving surrounding normal tissues, attributed to the low dose rate and limited radiation range [14, 15]. Conventionally, the risk of intestinal damage associated with ^125^I seed implantation has been regarded as substantially lower compared to that of external beam radiation. Nevertheless, with the accumulation of clinical experience, there is increasing evidence suggesting that radiation colitis is a noteworthy complication following ^125^I therapy, particularly in patients with tumors located in proximity to the bowel [3, 16]. This condition can result in chronic pain, diarrhea, bleeding, and even obstruction, significantly affecting long‐term quality of life and overall treatment outcomes [5, 17]. Consequently, elucidating the underlying pathogenesis of radiation colitis is of considerable clinical importance.

Currently, the comprehension of the pathogenesis of ^125^I‐induced radiation colitis is predominantly derived from an understanding of the direct biological effects of ionizing radiation. Radiation energy directly impacts intestinal epithelial stem cells and crypt cells, causing lethal DNA double‐strand breaks [18]. This damage subsequently initiates cell cycle arrest, mediated by factors such as p53/p21, which halts cells at the G1/S phase to facilitate repair [18, 19]. In cases where the damage is irreparable, cells undergo apoptosis, potentially via the Caspase‐3 pathway, or necrosis [20]. This direct cytotoxic effect disrupts the renewal cascade of the intestinal epithelium, leading to crypt loss, villous shortening, and impaired mucosal integrity, ultimately culminating in intestinal dysfunction and inflammation [8, 21]. In this study, including H&E staining that reveals submucosal separation and inflammatory cell infiltration, as well as TUNEL assays that confirm apoptosis in intestinal villous cells, strongly corroborate the involvement of this classical mechanism in ^125^I‐induced colitis.

In recent years, the role of gut microbiota in the pathogenesis of radiation colitis has garnered scholarly attention. Numerous studies have demonstrated that radiation exposure results in microbial diversity and structural dysbiosis, characterized by a decline in beneficial genera such as *Lactobacillus* and *Bifidobacterium*, alongside an increase in pathogenic bacteria like *Escherichia coli* and *Staphylococcus* [6, 22]. These microbial dysbiosis are intricately linked to the pathological progression of radiation colitis. For instance, the diminution of beneficial microbial metabolites, particularly short‐chain fatty acids (SCFAs) such as butyrate, undermines their critical roles in supplying energy to intestinal epithelial cells and inhibiting histone deacetylase activity, thereby impairing the processes of barrier repair [23]. Importantly, interventions such as fecal microbiota transplantation (FMT) aimed at augmenting the abundance of beneficial microbial families, including *Lactobacillaceae* and *Lachnospiraceae*, have been shown to elevate levels of protective metabolites, such as indole‐3‐carboxaldehyde and N‐acetyl serotonin, thereby mitigating the symptoms associated with radiation colitis [24]. Although *Bifidobacterium* is generally recognized as a beneficial commensal with anti‑inflammatory properties in many contexts, its increased abundance in our setting was associated with elevated levels of pro‑inflammatory cytokines and colonic tissue injury. Alternatively, the observed increase in *Bifidobacterium* could be attributed to its relatively higher tolerance to radiation‐induced oxidative stress compared to other highly sensitive obligate anaerobes. Consequently, the depletion of other major microbial taxa may have resulted in a mathematical or ecological expansion of *Bifidobacterium* in terms of relative abundance. Further absolute quantification studies are warranted to clarify this dynamic. Simultaneously, the proliferation of conditional pathogens can activate host Toll‐like receptor signaling pathways, particularly the TLR4‐MyD88 axis, thereby promoting the release of pro‐inflammatory cytokines such as IL‐1β, IL‐6, and TNF‐α [18, 25]. However, most studies focus on high‐dose, short‐term irradiation, with limited research on the impact of continuous low‐dose‐rate ^125^I irradiation on gut microbiota and its role in colitis development.

Our study addresses this gap by demonstrating that ^125^I irradiation significantly reduces SCFA‐producing taxa closely associated with gut health, such as *Lachnospiraceae_NK4A136_group* and *Lactobacillus*, consistent with trends observed in external beam irradiation studies. Correlation analysis demonstrated that the abundances of these taxa exhibited a significant negative correlation with the levels of pro‐inflammatory cytokines (IL‐1β, IL‐6) and a positive correlation with the expression of tight junction proteins (ZO‐1, Occludin). It is important to acknowledge that the correlation analyses conducted in this study are exploratory in nature and have not been adjusted for multiple comparisons. While this methodology enhances the sensitivity for identifying potential associations, it also increases the risk of type I errors. In Western blot analysis, one control sample showed relatively low expression of ZO‑1 and Occludin. This observation was reproducible across three independent experiments. We speculate that this individual mouse may have had undetected physiological variations contributing to lower baseline expression of tight junction proteins. The sample was retained in the analysis to reflect biological variation, and the overall statistical significance between groups remained unchanged with or without inclusion of this sample. Consequently, these findings should be interpreted with caution and considered as hypothesis‐generating. Future research with larger sample sizes and experimental validation, such as fecal microbiota transplantation and mono‐colonization with candidate taxa, will be crucial to substantiate the causal roles implied by these correlative data. RDA analysis identified IL‐1β and IL‐6 as the factors most closely associated with microbial changes, further linking dysbiosis to specific inflammatory pathways. These discoveries integrate the classical direct injury mechanisms of ^125^I‐induced colitis with the indirect mechanisms mediated by the microbiota. While these correlations suggest a potential protective role for these taxa, alternative explanations remain plausible. The observed microbial changes may be a direct consequence of radiation exposure, independent of their effects on host tissue; they could represent an epiphenomenon occurring concurrently with radiation‐induced injury; or they might result from, rather than cause, the inflammatory and barrier disruptions in the colon. Future studies utilizing fecal microbiota transplantation from irradiated mice into non‐irradiated recipients, or employing germ‐free mouse models, will be crucial to elucidate the causal relationships underlying these associations. Currently, the hypothesis that the depletion of beneficial bacteria contributes to ^125^I‐induced colitis should be regarded as a working model that requires experimental validation.

A note of caution is warranted regarding our use of the GMH and MDI. Both indices were originally developed using human datasets. Although GMHI was originally developed and validated using human microbiome datasets, its underlying principle—comparing the abundance of health associated versus disease associated taxa—is taxon based and not inherently species specific. The same approach can be applied to mouse studies because the index relies on taxonomic assignment at the species or genus level, which is consistently achievable in both human and murine microbiome data via reference databases. Moreover, GMHI and MDI has recently been employed in various diseases animal models [26, 27]. In the context of radiation colitis, we consider GMHI and MDI appropriate because they provide a holistic, data driven assessment of microbial health and dysbiosis, which complements traditional diversity metrics and helps link microbial alterations to disease phenotype. It is well established that many bacteria exhibit context‑dependent effects that differ between species; examples include *Citrobacter rodentium* and *Helicobacter hepaticus*, which are pathogenic in mice but not in humans, as well as *Akkermansia muciniphila*, whose beneficial effects vary across hosts and disease contexts. Moreover, mouse models of human diseases often do not fully recapitulate the microbiome features observed in human patients. Therefore, while GMHI and MDI were technically applicable to our dataset, we emphasize that their use in this study was strictly exploratory and intended to complement. Future studies employing disease‑specific indices or directly validating GMHI/MDI in murine contexts will be necessary to establish their applicability.

In addition, we conducted transcriptomic analyses to identify differentially expressed genes and molecular signaling pathways linked to alterations in gut microbiota. Prior research has demonstrated that the chemokines Ccl7 and Cxcl14 are significantly upregulated in colonic inflammation [28, 29]. These molecules play a crucial role in recruiting monocytes and macrophages, thereby facilitating inflammatory cell infiltration and exacerbating intestinal damage [30]. Simultaneously, C7 as a component of the complement system, contributes to intestinal pathology through excessive activation, leading to epithelial cell lysis—a mechanism implicated in the tissue damage observed in Crohn's disease and necrotizing enterocolitis [30]. Furthermore, the elevation of Apoh, associated with intestinal ischemia, may contribute to microthrombosis‐induced intestinal necrosis [31, 32]. The suppressor Cish plays a critical role in the negative regulation of the JAK‐STAT signaling pathway and affects the survival of intraepithelial lymphocytes; its absence has been shown to exacerbate intestinal inflammation [33, 34]. Concurrently, Angptl7, recognized as an angiogenic factor, is potentially implicated in vascular remodeling during the repair of intestinal injuries [35]. In our study, significant alterations in these key factors suggest that they may serve as essential targets through which the gut microbiota impacts colonic pathophysiology.

Furthermore, the KEGG pathway analysis identified significant enrichment in the “Cytokine‐cytokine receptor interaction” and “IL‐17 signaling pathway.” These pathways are well‐recognized for their central roles in the recruitment of immune cells and the mediation of inflammation, acting as crucial links between gut microbiota dysbiosis and tissue inflammation [36, 37]. While these pathways are well‐known mediators of inflammation and immune responses, we emphasize that these findings are derived from transcriptomic predictions rather than direct functional validation. The enrichment analysis reflects coordinate changes in gene expression that are consistent with activation of these pathways, but experimental approaches—such as blockade of IL‐17 signaling or measurement of downstream effector proteins—would be required to confirm their actual involvement in ^125^I‐induced colitis. Therefore, we present these pathways as candidate mechanisms that may contribute to the pathogenesis of radiation‐induced intestinal injury, rather than as definitive conclusions. These hypotheses will guide our future mechanistic investigations. Of particular interest was the differential expression of numerous immunoglobulin variable region genes, such as “Igkv6‐17” and “Igkv1‐132” [38]. As vital components of B cell receptors, the collective upregulation of these genes suggests that ^125^I irradiation may activate local humoral immune responses within the intestinal mucosa [39]. This observation implies that B cells and their antibody products may play previously underappreciated roles in antigen clearance, immune complex formation, or regulatory functions within the microenvironment of radiation‐induced intestinal damage [37]. Furthermore, Procrustes analysis confirmed a coordinated variation between these differentially expressed genes and microbial communities, demonstrating that host transcriptional responses and structural changes in the gut microbiota form an integrated molecular network in ^125^I‐induced colitis.

This study has several limitations. The inflammatory response is currently based on mRNA expression of pro‐inflammatory cytokines (IL‐1α, IL‐1β, IL‐6, and TNF‐α), without corresponding protein‐level validation. While these transcriptional changes provide valuable initial evidence of immune activation, we recognize that cytokine secretion and immune cell infiltration—key hallmarks of active inflammation—require direct confirmation. While we found strong correlations between microbiota, gene expression, and phenotypic outcomes, causality is not established. Future validation with fecal microbiota transplants or germ‐free models is needed. The roles of key microbial taxa and host genes require further study through in vitro co‐culture systems or gene knockout models to clarify their roles in radiation colitis. Notably, we acknowledge the relatively small sample size (*n* = 5 per group) as a limitation, which may affect the statistical power of our microbiome and correlation analyses, and future studies with larger cohorts are needed to validate our findings. Our study was limited to one observational timepoint, preventing a dynamic analysis of changes post‐^125^I exposure. Additionally, our findings are based on a controlled animal model with a small sample size.

## 4 Conclusions

This study explores radiation colitis after ^125^I therapy using multi‐omics analysis, revealing significant alterations in the gut microbiota that are closely associated with the pathogenesis of this condition. These findings expand the current understanding beyond direct radiation‐induced injury to encompass microecological perturbations. These findings establish a foundational basis for future research into microbiome‐based therapeutic strategies; however, further validation is necessary to confirm the causal role of the microbiota in this process.

## 5 Materials and Methods

### 5.1 Ethics Statement

All experimental procedures involving animals were conducted in compliance with the National Institutes of Health Guide for the Care and Use of Laboratory Animals and received approval from the local Animal Care and Use Committee at Army Medical University (Approval number: AMUWEC20217036).

### 5.2 ^125^I Seeds

The ^125^I seed (batch no. 191025), manufactured by Tianjin Saide Biology Pharmacy Co. Ltd, Tianjin, China, was supplied by the Seed Therapy Center at the Tumor Hospital of Shaanxi Province. The seed features a diameter of 0.8 mm and a length of 4.5 mm, with an average energy range of 27.4–35.5 keV and a half‐life of 59.6 days. The activity of an individual ^125^I seed is 0.7 mCi, and it exhibits an initial dose rate of 5.13 cGy/h.

### 5.3 Mice Models

Six‐ to eight‐week‐old specific pathogen‐free male C57BL/6 J mice were procured from GemPharmatech (Chengdu, China) and housed at the animal facility. The mice were maintained under humane conditions, with a 12‐h light/dark cycle and ad libitum access to food and water. Following a 7‐day acclimatization period with co‐housing, the mice were randomly assigned to control or ^125^I implantation groups using a random number table. Throughout the subsequent experimental period, the two groups were maintained in separate cages, and the treatments and measurements were performed in random order. Group allocation was performed by an investigator not involved in subsequent procedures. Blinding was maintained during all experimental procedures, outcome assessments, and data analysis. Both groups of mice were anesthetized via intraperitoneal injection with approximately 100–150 µL of 2.5% 2,2,2‐Tribromoethanol (Sigma, USA). An abdominal laparotomy was performed to expose the colon, and radioactive ^125^I seeds were implanted near the descending colon of the lower left abdomen using an 18 G implant needle. After a period of 2 weeks, fecal samples were collected, and colonic tissues were harvested (*n* = 5 per group, with three independent replicates; sample size based on pilot experiments and published literature). All mice were healthy at the time of randomization, and no a priori exclusion criteria were established. No animals were excluded during the experiment, and all collected data points were included in the final analysis. The outcome measures were histological scores (H&E and TUNEL), pro‑inflammatory cytokines (IL‑1α, IL‑1β, IL‑6, TNF‑α) and tight junction proteins (ZO‑1, occludin). Sample size was enough to provide sufficient power to detect biologically meaningful differences in the outcome measures.

### 5.4 16S rRNA Gene Sequencing

Fecal DNA was extracted utilizing a DNA extraction kit (Mabio, Guangzhou, China). PCR amplification was conducted with universal amplicon primers targeting the V4 region of the bacterial 16S rRNA gene (forward primer: 5’‐GTGTGYCAGCMGCCGCGGTAA‐3’; reverse primer: 5’‐CCGGACTACNVGGGTWTCTAAT‐3’), in conjunction with the extracted fecal DNA and a fluorescent dye. The resulting PCR products underwent analysis via Illumina high‐throughput sequencing on the NovaSeq. 6000 platform. Subsequently, QIIME2 was employed for data extraction, sequence assembly, and comprehensive bioinformatics analysis. Alpha diversity indices were compared between the control and ^125^I‐treated groups using the Wilcoxon rank‐sum test. Beta diversity was assessed using principal coordinate analysis (PCoA) and non‐metric multidimensional scaling (NMDS) based on Bray–Curtis dissimilarity. Significant differences in microbial community structure between the control and ^125^I‐treated groups were evaluated using analysis of similarities (ANOSIM) with 999 permutations. *p* < 0.05 were considered statistically significant.

### 5.5 RNA‐Seq Analysis

For RNA sequencing, total RNA was extracted from colonic tissues using the Trizol reagent (Takara, Tokyo, Japan) in accordance with the manufacturer's instructions. The concentration of the extracted RNA was measured with a NanoDrop 2000 Spectrophotometer (Thermo Scientific, MA, USA). Subsequently, RNA isolation was performed using the NEBNext Poly(A) mRNA Magnetic Isolation Module. The mRNA library was then constructed using the NEBNext Ultra II mRNA Library Prep Kit for Illumina. Library concentration was assessed with the Qubit dsDNA HS Assay Kit, and sequencing was conducted on the NovaSeq. 6000 platform (Illumina, USA).

### 5.6 qRT‐PCR Analyses

Total RNA was isolated from tissue samples utilizing the TRIzol reagent. Subsequently, complementary DNA (cDNA) was synthesized and combined with SYBR Green Master Mix (Promega, Madison, WI, USA) and gene‐specific primers. Quantitative reverse‐transcription PCR (qRT‐PCR) was conducted using the 7500 Real‐Time PCR System (Applied Biosystems, MA, USA). Gene expression alterations were quantified employing the ΔΔCT method. The sequences of the specific primers utilized are detailed in Table S1.

### 5.7 Histopathological Examination

Colonic tissues were preserved in a 4% paraformaldehyde solution (Sangon Biotech, China). Histological damage within the mucosa, stained with hematoxylin and eosin (H&E), was assessed through quantitative measurement of tissue injury by an observer blinded to the experimental conditions. The evaluation of damage in the colonic tissue samples was conducted as previously documented [40].

### 5.8 TUNEL Assay

The TUNEL assay was conducted on paraffin‐embedded slides using the DAB (SA‐HRP) TUNEL Apoptosis Detection Kit (G1507; Servicebio, China), in accordance with the manufacturer's protocol.

### 5.9 Western Blot Assay

Briefly, total protein was extracted using RIPA lysis buffer (Solarbio, China) with 1% phenylmethanesulfonyl fluoride (Boster, China), followed by centrifugation at 12,000 g for 10 min at 4°C. The protein was separated via SDS‐PAGE and transferred to a polyvinylidene fluoride membrane. Blots were blocked with QuickBlock Western blocking solution (Beyotime, China) and incubated overnight at 4°C with anti‐occludin, anti‐ZO1, and anti‐GAPDH antibodies (all 1:1000; Servicebio, China). Afterward, they were incubated with a horseradish peroxidase‐conjugated secondary antibody (1:2000; Proteintech, China) for 1 h at room temperature. Detection was performed using a chemiluminescent system and quantified with Image J software.

### 5.10 Quantification and Statistical Analyses

Measurement data following a normal distribution are reported as the mean ± standard deviation (SD). Statistical significance between two groups was evaluated using a two‐tailed unpaired Student's *t*‐test. For comparisons involving multiple datasets, a one‐way analysis of variance (ANOVA) followed by Tukey's post‐hoc test was employed. All statistical analyses were conducted using GraphPad Prism 9.0, unless specified otherwise. Data normality was assessed using normality and log‐normality tests. For data that did not meet normality assumptions, non‑parametric tests (Mann–Whitney U test for two‑group comparisons, and Kruskal–Wallis test for multiple comparisons) were applied. Statistical significance is denoted by p‐values as follows: *p < 0.05, **p < 0.01, ***p < 0.001, with “ns” indicating no significance.

## Author Contributions

**Pingping Liu:** writing – original draft, investigation, methodology. **Xiong Zeng:** methodology, investigation, writing – original draft. **Wenjing Liu:** data curation, formal analysis. **Huichao Xie:** methodology, investigation. **Yongkun Fang:** visualization. **Erwan Yang:** software, formal analysis. **Xiaoqi Tang:** resources, supervision. **Chenglin Fan:** project administration. **Yihui Chen:** conceptualization, funding acquisition, writing – review and editing, validation.

## Conflicts of Interest

The authors declare no conflicts of interest.

## Supporting information


Supporting File 1

Supporting File 2

Supporting File 3

Supporting File 4

Supporting File 5

Supporting File 6

Supporting File 7

Supporting File 8

Supporting File 9

Supporting File 10

## Data Availability

The data that support the findings of this study are available from the corresponding author upon reasonable request. The data that support the findings of this study are available from the corresponding author upon reasonable request. The raw sequencing reads were deposited into the NCBI Sequence Read Archive (SRA) database (Accession Number: PRJNA1440933).
