# Supplementary material for: Gut Microbiota Dysbiosis Mediates 125I‐Induced Radiation Colitis via Host Transcriptional Regulation
Source: Immun Inflamm Dis. 2026 Sep 15;14(9):e70523. doi: 10.1002/iid3.70523 (PMC13576935; doi:10.1002/iid3.70523)

Spearman Correlation Heatmap

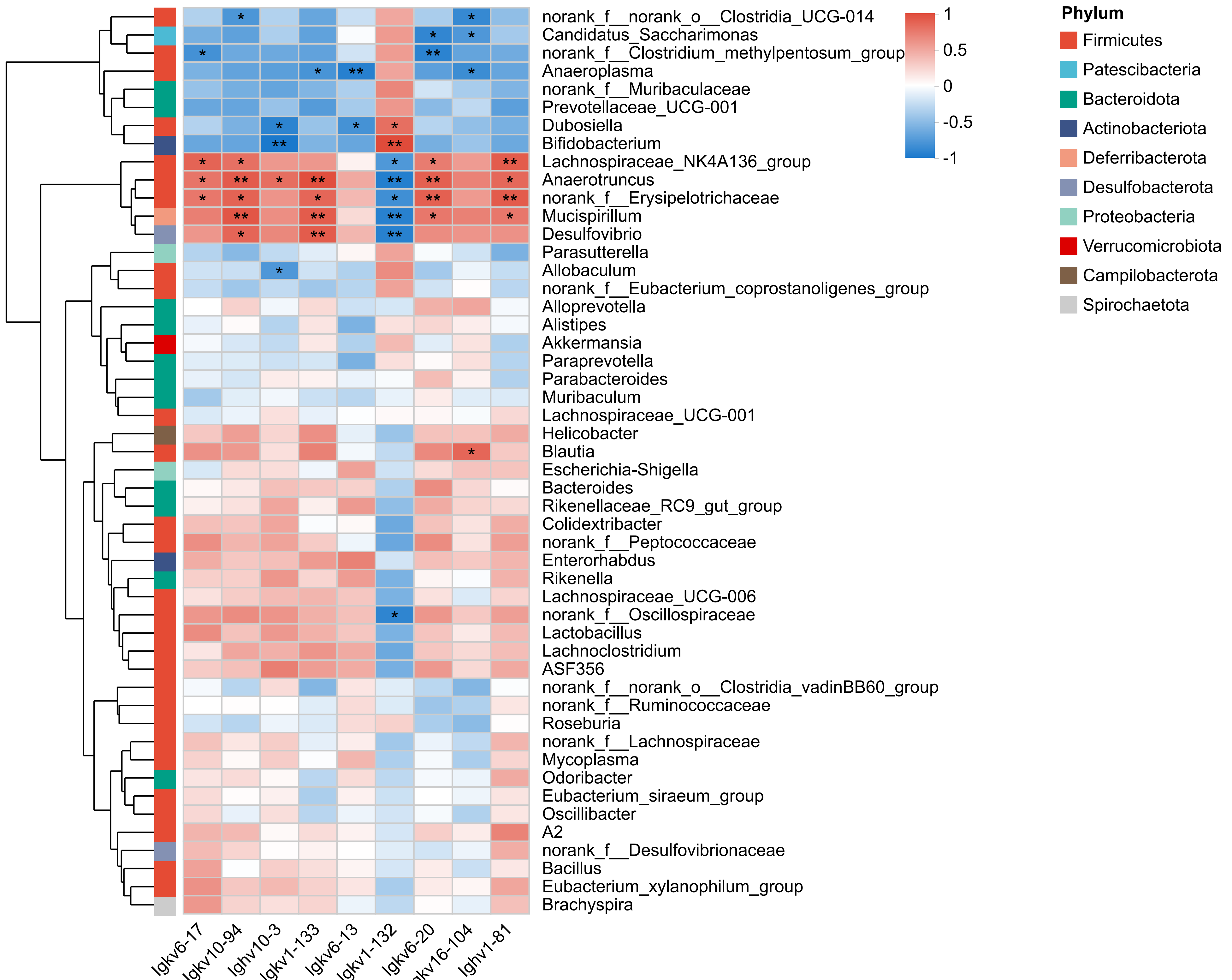

Supplement: Supplementary file 2 [file IID3-14-e70523-s004.pdf]
