# Supplementary material for: Gut Microbiota Dysbiosis Mediates 125I‐Induced Radiation Colitis via Host Transcriptional Regulation
Source: Immun Inflamm Dis. 2026 Sep 15;14(9):e70523. doi: 10.1002/iid3.70523 (PMC13576935; doi:10.1002/iid3.70523)
Supplement: Supplementary file 3 [file IID3-14-e70523-s006.pdf]

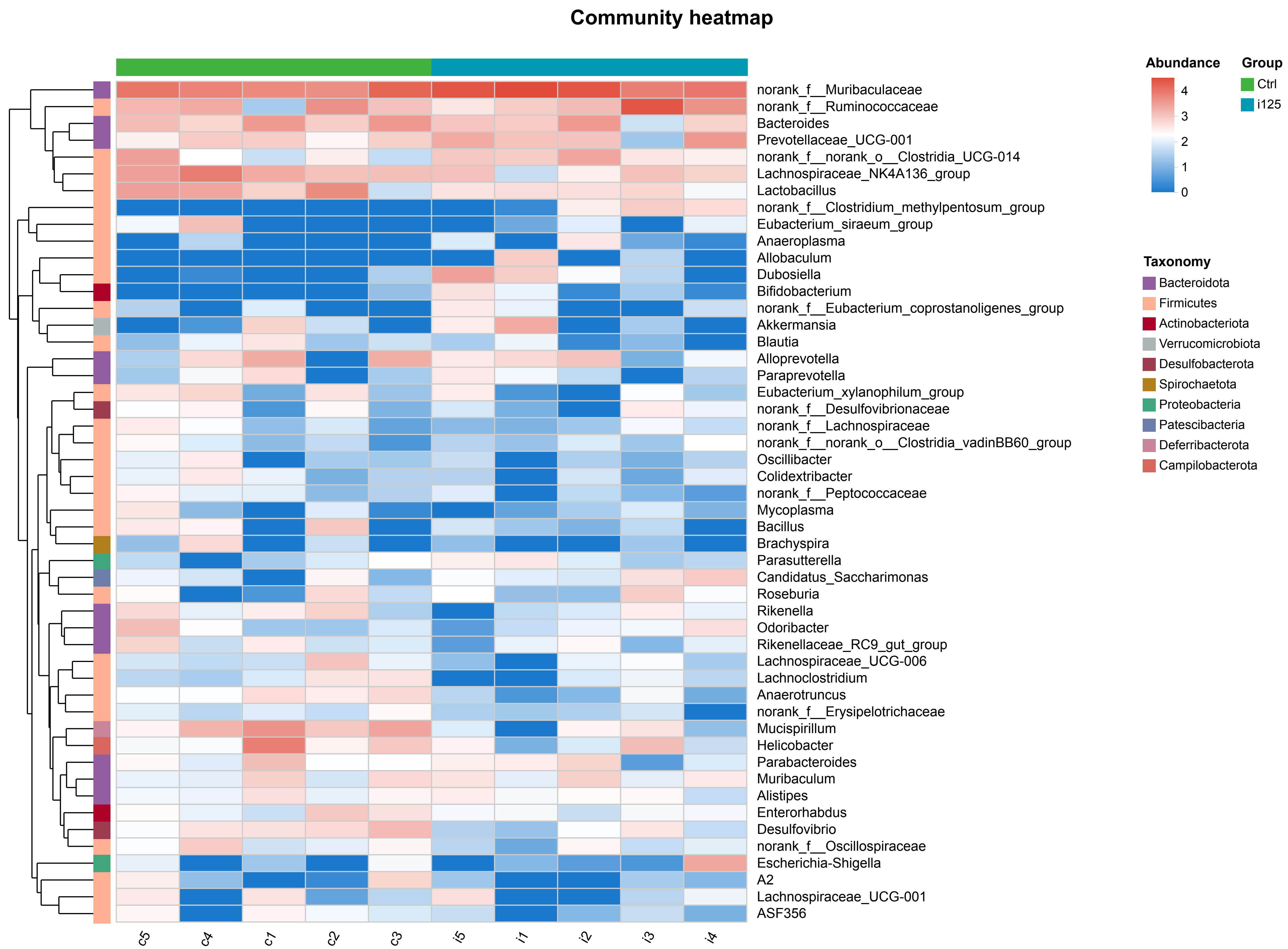

Community heatmap
Abundance
4
3
2
1
0
Group
Ctrl
i125
Taxonomy
Bacteroidota
Firmicutes
Actinobacteriota
Verrucomicrobiota
Desulfobacterota
Spirochaetota
Proteobacteria
Patescibacteria
Deferribacterota
Campilobacterota
norank_f__Muribaculaceae
norank_f__Ruminococcaceae
Bacteroides
Prevotellaceae_UCG-001
norank_f__norank_o__Clostridia_UCG-014
Lachnospiraceae_NK4A136_group
Lactobacillus
norank_f__Clostridium_methylpentosum_group
Eubacterium_siraeum_group
Anaeroplasma
Allobaculum
Dubosiella
Bifidobacterium
norank_f__Eubacterium_coprostanoligenes_group
Akkermansia
Blautia
Alloprevotella
Paraprevotella
Eubacterium_xylanophilum_group
norank_f__Desulfovibrionaceae
norank_f__Lachnospiraceae
norank_f__norank_o__Clostridia_vadinBB60_group
Oscillibacter
Colidextribacter
norank_f__Peptococcaceae
Mycoplasma
Bacillus
Brachyspira
Parasutterella
Candidatus_Saccharimonas
Roseburia
Rikenella
Odoribacter
Rikenellaceae_RC9_gut_group
Lachnospiraceae_UCG-006
Lachnoclostridium
Anaerotruncus
norank_f__Erysipelotrichaceae
Mucispirillum
Helicobacter
Parabacteroides
Muribaculum
Alistipes
Enterorhabdus
Desulfovibrio
norank_f__Oscillospiraceae
Escherichia-Shigella
A2
Lachnospiraceae_UCG-001
ASF356
c5
c4
c1
c2
c3
i5
i1
i2
i3
i4
