# Supplementary material for: Gut Microbiota Dysbiosis Mediates 125I‐Induced Radiation Colitis via Host Transcriptional Regulation
Source: Immun Inflamm Dis. 2026 Sep 15;14(9):e70523. doi: 10.1002/iid3.70523 (PMC13576935; doi:10.1002/iid3.70523)
Supplement: Supplementary file 4 [file IID3-14-e70523-s007.pdf]

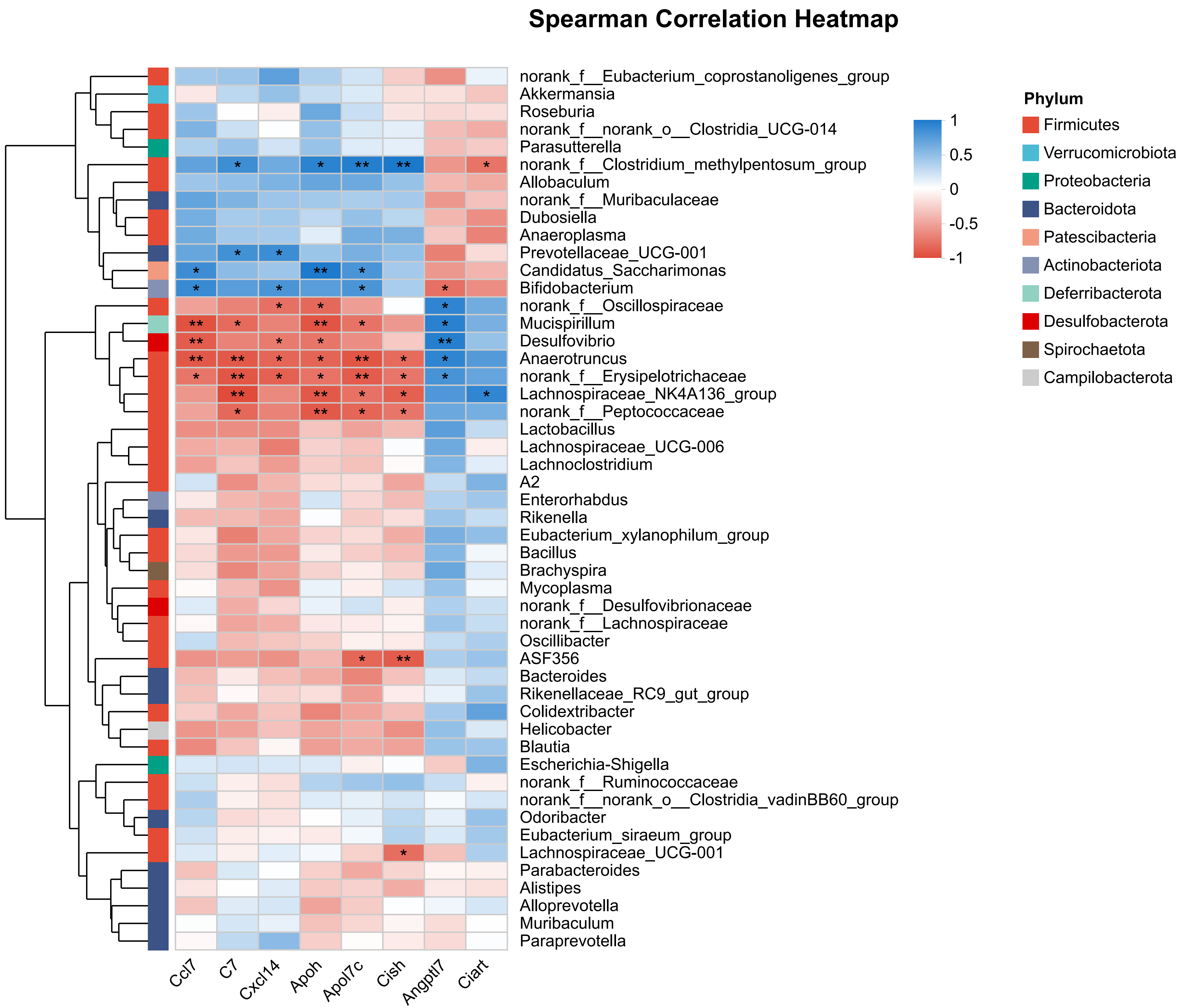
Spearman Correlation Heatmap
norank_f__Eubacterium_coprostanoligenes_group
Akkermansia
Roseburia
norank_f__norank_o__Clostridia_UCG-014
Parasutterella
norank_f__Clostridium_methylpentosum_group
Allobaculum
norank_f__Muribaculaceae
Dubosiella
Anaeroplasma
Prevotellaceae_UCG-001
Candidatus_Saccharimonas
Bifidobacterium
norank_f__Oscillospiraceae
Mucispirillum
Desulfovibrio
Anaerotruncus
norank_f__Erysipelotrichaceae
Lachnospiraceae_NK4A136_group
norank_f__Peptococcaceae
Lactobacillus
Lachnospiraceae_UCG-006
Lachnoclostridium
A2
Enterorhabdus
Rikenella
Eubacterium_xylanophilum_group
Bacillus
Brachyspira
Mycoplasma
norank_f__Desulfovibrionaceae
norank_f__Lachnospiraceae
Oscillibacter
ASF356
Bacteroides
Rikenellaceae_RC9_gut_group
Colidextribacter
Helicobacter
Blautia
Escherichia-Shigella
norank_f__Ruminococcaceae
norank_f__norank_o__Clostridia_vadinBB60_group
Odoribacter
Eubacterium_siraeum_group
Lachnospiraceae_UCG-001
Parabacteroides
Alistipes
Alloprevotella
Muribaculum
Paraprevotella
Ccl7
C7
Cxcl14
Apoh
Apol7c
Cish
Angptl7
Ciart
1
0.5
0
-0.5
-1
Phylum
Firmicutes
Verrucomicrobiota
Proteobacteria
Bacteroidota
Patescibacteria
Actinobacteriota
Deferribacterota
Desulfobacterota
Spirochaetota
Campilobacterota
