# Supplementary material for: Gut Microbiota Dysbiosis Mediates 125I‐Induced Radiation Colitis via Host Transcriptional Regulation
Source: Immun Inflamm Dis. 2026 Sep 15;14(9):e70523. doi: 10.1002/iid3.70523 (PMC13576935; doi:10.1002/iid3.70523)
Supplement: Supplementary file 5 [file IID3-14-e70523-s008.pdf]

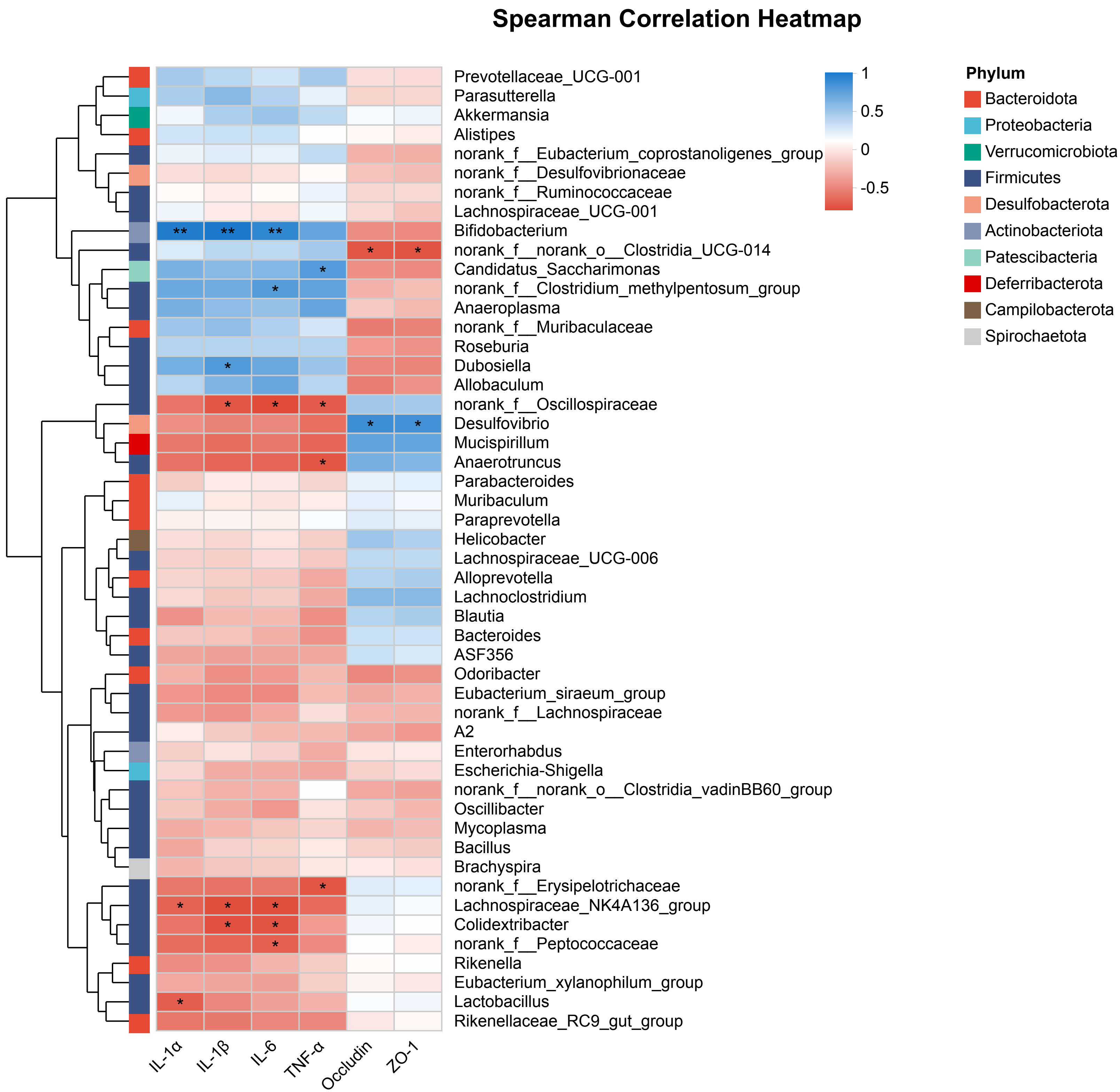

Spearman Correlation Heatmap
1
0.5
0
-0.5
Phylum
Bacteroidota
Proteobacteria
Verrucomicrobiota
Firmicutes
Desulfobacterota
Actinobacteriota
Patescibacteria
Deferribacterota
Campilobacterota
Spirochaetota
Prevotellaceae_UCG-001
Parasutterella
Akkermansia
Alistipes
norank_f__Eubacterium_coprostanoligenes_group
norank_f__Desulfovibrionaceae
norank_f__Ruminococcaceae
Lachnospiraceae_UCG-001
Bifidobacterium
norank_f__norank_o__Clostridia_UCG-014
Candidatus_Saccharimonas
norank_f__Clostridium_methylpentosum_group
Anaeroplasma
norank_f__Muribaculaceae
Roseburia
Dubosiella
Allobaculum
norank_f__Oscillospiraceae
Desulfovibrio
Mucispirillum
Anaerotruncus
Parabacteroides
Muribaculum
Paraprevotella
Helicobacter
Lachnospiraceae_UCG-006
Alloprevotella
Lachnoclostridium
Blautia
Bacteroides
ASF356
Odoribacter
Eubacterium_siraeum_group
norank_f__Lachnospiraceae
A2
Enterorhabdus
Escherichia-Shigella
norank_f__norank_o__Clostridia_vadinBB60_group
Oscillibacter
Mycoplasma
Bacillus
Brachyspira
norank_f__Erysipelotrichaceae
Lachnospiraceae_NK4A136_group
Colidextribacter
norank_f__Peptococcaceae
Rikenella
Eubacterium_xylanophilum_group
Lactobacillus
Rikenellaceae_RC9_gut_group
IL-1α
IL-1β
IL-6
TNF-α
Occludin
ZO-1
