# Supplementary material for: Gut Microbiota Dysbiosis Mediates 125I‐Induced Radiation Colitis via Host Transcriptional Regulation
Source: Immun Inflamm Dis. 2026 Sep 15;14(9):e70523. doi: 10.1002/iid3.70523 (PMC13576935; doi:10.1002/iid3.70523)
Supplement: Supplementary file 6 [file IID3-14-e70523-s005.docx]

**Table S1. List of DNA sequences**

| **DNA Name** | **Sequence** |
| --- | --- |
| Actb | F: GGCTGTATTCCCCTCCATCG |
|  | R: CCAGTTGGTAACAATGCCATGT |
| Occludin | F: TTGAAAGTCCACCTCCTTACAGA |
|  | R: CCGGATAAAAAGAGTACGCTGG |
| Claudin-1 | F: GGGGACAACATCGTGACCG |
|  | R: AGGAGTCGAAGACTTTGCACT |
| Claudin-2 | F: CAACTGGTGGGCTACATCCTA |
|  | R: CCCTTGGAAAAGCCAACCG |
| ZO-1 | F: GCCGCTAAGAGCACAGCAA |
|  | R: TCCCCACTCTGAAAATGAGGA |
| IL-1α | F: GCACCTTACACCTACCAGAGT |
|  | R:AAACTTCTGCCTGACGAGCTT |
| IL-1β | F: GCAACTGTTCCTGAACTCAACT |
|  | R: ATCTTTTGGGGTCCGTCAACT |
| IL-6 | F: TAGTCCTTCCTACCCCAATTTCC |
|  | R:TTGGTCCTTAGCCACTCCTTC |
| IL-10 | F: CTTACTGACTGGCATGAGGATCA |
|  | R:GCAGCTCTAGGAGCATGTGG |
| TNF-α | F: AGGGTCTGGGCCATAGAACT |
|  | R:CCACCACGCTCTTCTGTCTAC |
| Angptl7 | F: TGACTGTTCTTCCCTGTACCA |
|  | R:CAAGGCCACTCTTACGTCTCT |
| Apoh | F: TGCCATGTTGCTATTGCAGGA |
|  | R:GGCTTGCAGGAGTAGACAATCT |
| C7 | F: TTGCTGGATGAGTTAATGGGC |
|  | R:GTATTTGTGAACAATTCCGCAGG |
| Ccl7 | F: GCTGCTTTCAGCATCCAAGTG |
|  | R:CCAGGGACACCGACTACTG |
| Ciart | F: CTGAACGGACTCAAGATGGGT |
|  | R:ACCTCCTGAGGATGACTTCTG |
| Cish | F: ATGGTCCTTTGCGTACAGGG |
|  | R:GGAATGCCCCAGTGGGTAAG |
| Cxcl14 | F: GAAGATGGTTATCGTCACCACC |
|  | R:CGTTCCAGGCATTGTACCACT |
